# Trend and burden of lower-limb amputations due to diabetes: time series analysis, Amazonas, 2012–2021

**DOI:** 10.1590/S2237-96222026v35e20250534.en

**Published:** 2026-03-16

**Authors:** Cilio Antonio Ribeiro, Sabrina da Silva Santos, Thatiana Lameira Maciel Amaral

**Affiliations:** 1 Fundação Oswaldo Cruz, Escola Nacional de Saúde Pública Sergio Arouca, Rio de Janeiro, RJ, Brazil; 2 Universidade Federal do Acre, Rio Branco, AC, Brazil

**Keywords:** Diabetes Mellitus, Surgical, Amputation, Time Series Studies, Global Burden of Disease, Disability-Adjusted Life Years, Diabetes Mellitus, Amputación Quirúrgica, Estudios de Series Temporales, Carga Global de Enfermedades, Años de Vida Ajustados por Discapacidad

## Abstract

**Objective:**

To calculate the temporal trend and the burden of disease for lower-limb amputations due to diabetes in Amazonas, Brazil, from 2012 to 2021.

**Methods:**

This is a time series analysis study using data from the Hospital Information System of the Brazilian Unified Health System. Specific and age-adjusted rates, the annual percent change of amputations, and the 95% confidence interval were calculated for the period 2012–2021. Years lived with disability were calculated and stratified by sex and age group between 2017 and 2021.

**Results:**

During the 10 years analyzed, 3,621 lower-limb amputation procedures were performed in people with diabetes in public hospitals in Amazonas. An increase in age-adjusted amputation rates among men was observed, rising from 8.33 per 100 thousand men in 2012 to 15.55 per 100 thousand men in 2021, with an annual percent change of 10.5% and a 95% confidence interval between 3.8% and 17.6%. The disease burden totaled 21,845.11 years lived with disability, of which 70.4% corresponded to men. Most years lived with disability were concentrated in the 45–64 age group (58.2%).

**Conclusion:**

There was a male predominance in the increasing trend of lower-limb amputations and in the high burden of disease in Amazonas.

## Resumen

**Objetivo:**

Calcular la tendencia temporal y la carga de enfermedad de las amputaciones de miembros inferiores por diabetes en el estado de Amazonas, Brasil, en el período 2012-2021.

**Métodos:**

Se trata de un estudio de análisis de serie temporal con datos del Sistema de Información Hospitalaria del Sistema Único de Salud. Se calcularon las tasas específicas y ajustadas por edad, la variación porcentual anual de las amputaciones y el intervalo de confianza del 95% en el período 2012-2021. Se calcularon los años vividos con discapacidad, estratificados por sexo y grupo etario, entre 2017 y 2021.

**Resultados:**

Durante los 10 años analizados, se realizaron 3.621 procedimientos de amputación de miembros inferiores en personas con diabetes en los hospitales públicos del estado de Amazonas. Se verificó un aumento de las tasas de amputación ajustadas por edad en hombres, pasando de 8,33 en 2012 a 15,55 por 100 mil hombres en 2021, con una variación porcentual anual de 10,5% y un intervalo de confianza del 95% entre 3,8% y 17,6%. La carga de enfermedad totalizó 21.845,11 años vividos con discapacidad, de los cuales el 70,4% correspondió a los hombres. La mayor parte de los años vividos con discapacidad se concentró entre los 45 y 64 años (58,2%).

**Conclusión:**

Hubo predominancia masculina en la tendencia creciente de las amputaciones de miembros inferiores y en la elevada carga de enfermedad en el estado de Amazonas.

**Ethical aspects**

This study used publicly available, anonymized databases with unrestricted access from the Brazilian Unified Health System Information Technology Department (DATASUS).

## Introduction 

Chronic noncommunicable diseases constitute the main cause of morbidity and mortality worldwide, with a greater effect on the most vulnerable populations living in low- and middle-income countries (1). Among these diseases, diabetes imposes a significant economic and social burden on individuals and health systems, as it is associated with chronic complications of varying severity that impair functional capacity, reduce autonomy, and affect quality of life (2,3).

Lower-limb complications related to diabetes often begin with neuropathy, a critical risk factor for the development of ulceration, which in turn increases susceptibility to infections and amputations (4,5). About 90% of non-traumatic lower-limb amputations were attributed to diabetes between 2003 and 2013. Globally, every 30 seconds, a lower limb is amputated due to complications associated with the disease, and more than 50% of people with diabetes die within five years after the first amputation (4,6).

In 2019, 606,785 adults with diabetes (6%) reported ulcers or amputations in Brazil (7). In Amazonas, the incidence of diabetes was nearly twice as high among women (165.23/100 thousand inhabitants) compared to men (93.72/100 thousand inhabitants) in 2013. However, more severe chronic complications, such as lower-limb amputations, showed higher incidences in males (4.99/100 thousand inhabitants) compared to females (2.75/100 thousand inhabitants). 

In Brazil and Amazonas, 55% of people with diabetes had never had their feet examined by a health professional. The lack of continuous follow-up was also evident in glycemic control monitoring: in 2013, 10% of people with diabetes in the country had lived with the disease for more than a year without regular blood glucose measurement. In Amazonas, this figure rose to 12% (8). These weaknesses in care and the divergence in the epidemiological profile between men and women reinforce the importance of investigations tailored to the local context and analyses stratified by sex.

Given the increase in life expectancy and the high prevalence of chronic noncommunicable diseases, measurement of population health status should account for temporal trends and the time lived with a given disease (1,9). The World Health Organization (WHO) adopted years lived with disability as an important indicator of the magnitude of the global burden of disease, aiming to measure the impact of morbidity on population health, be sensitive to health disparities, and be useful for identifying vulnerable groups (10). In 2021, it was reported that years lived with disability due to diabetes increased in all countries, with a global rise of 26% between 2010 and 2021 (11,12).

This article aimed to calculate the temporal trend and disease burden for lower-limb amputations due to diabetes in the general population of Amazonas from 2012 to 2021.

## Methods 

### Study design

This is a time series analysis and disease burden study of lower-limb amputations due to diabetes in the general population of Amazonas. The years lived with disability (YLD) indicator was used, a measure that captures the effect of morbidity on population health status.

### Setting 

Amazonas is located in the Northern region and is the largest state in the country in territorial extension, with an area of 1,559,255.881 km^2^. According to the 2022 Demographic Census of the Brazilian Institute of Geography and Statistics (*Instituto Brasileiro de Geografia e Estatística*, IBGE), the state’s population reached 3,941,613 inhabitants in 2022. The population density was 2.53 inhabitants/km^2^, making it the second most populous state in the North (13). 

Amazonas is composed of 62 municipalities. For analytical purposes, this study adopted the geographic stratification based on the four intermediate geographic regions defined by IBGE: Lábrea, Manaus, Parintins, and Tefé.

### Participants 

The study covered the general population of Amazonas, of both sexes and all ages, from 2012 to 2021. In this population, the total number of amputations among individuals with diabetes was identified, based on the municipality of residence of the amputees, recorded in the Hospital Information System database of the Brazilian Unified Health System (*Sistema de Informações Hospitalares do Sistema Único de Saúde*, SIH/SUS).

### Variables and data sources

Information on amputations was obtained from the SIH/SUS (14), based on hospital admission authorizations of individuals undergoing amputation procedures due to diabetes in Amazonas, from 2012 to 2021, available from the DATASUS. 

The data necessary to identify the total number of amputation procedures were: primary diagnosis (International Statistical Classification of Diseases and Related Health Problems 10th Revision: E10–E14), sex, age group at the date of amputation, municipality of residence in Amazonas for determining intermediate geographic regions, and procedures performed according to level of amputation (major lower limbs, foot/tarsus, toe) (15).

The populations of men and women residing in Amazonas, by age group, were obtained from the DATASUS database (https://datasus.saude.gov.br/), using the study on population estimates by municipality, age, and sex (16) for the period 2012–2021. These populations were used to calculate the rates of amputation procedures.

### Bias control

The collected data refer to hospital admission authorizations for amputation surgeries due to diabetes. The distributions and rates of amputations presented should be interpreted as the number and magnitude of procedures performed, not as the number of individuals undergoing amputations or incidence rates.

For YLD calculations, incident case estimates were used. Given the absence of this parameter for Amazonas, the incidence of amputations was estimated considering a mean reamputation rate of 45.7% (17). The number of hospital admission authorizations was divided by 1.457.

### Statistical methods

The data were organized in Microsoft Excel spreadsheets and presented as absolute and proportional values by sex, age group, and intermediate geographic regions of Amazonas.

For the temporal trend, specific and age-standardized rates of lower-limb amputations due to diabetes were calculated. For this age standardization, the direct method was used, with the standard population being the average population of men and women in Amazonas between 2012 and 2021 (16). This period was selected because it provided a consistent historical series, with more standardized data in the SIH/SUS from 2012 onward, thereby increasing the comparability and reliability of the records.

The Joinpoint Trend Analysis Software, version 4.9.1.0 (available at https://surveillance.cancer.gov/joinpoint/), was used to determine the annual percent change (APC) and the 95% confidence interval (95%CI) of the age-standardized amputation rates, which were calculated by the program. The parameters considered in the trend analysis included: rates as the dependent variable, year as the independent variable, a minimum of 0 and a maximum of 5 joinpoints, adjustment of errors for first-order autocorrelation calculated from the data, Bayesian Information Criterion (BIC) for the selection of the final model, and a significance level of 5%.

For the analysis of the disease burden associated with diabetes-related amputation, YLD was calculated for the period 2017–2021 as follows: YLD=I×P×D, where I corresponded to the total number of incident amputation cases; P referred to the disability weight; and D represented the average duration of permanent disability resulting from the amputation (considered until the end of the individual’s life expectancy) (10). 

A life expectancy at birth of 89.95 years (expected age at death) was considered, according to the theoretical minimum risk life table from the 2021 Global Burden of Disease (GBD) study (12). The disability weight used in this study was 0.36, corresponding to the weight assigned to amputations according to Murray and Lopez (18). They quantified the health loss occurring during the time lived with the disease, ranging from 0 (full health) to 1 (worst health, equivalent to death).

Absolute and proportional YLD values were presented. One YLD represented one year of healthy life lost due to disability (10). Unadjusted YLD rates per 100 thousand men and women in the Amazonas population were presented for each age group, along with age-adjusted YLD rates, using the standard global population from the 2021 Global Burden of Disease (GBD) study (11).

## Results 

During the 10 years analyzed (2012–2021), 3,621 lower-limb amputations were performed in public hospitals in Amazonas, distributed across four intermediate geographic regions. The majority of amputations occurred in men (68.1%) (Table 1).

The mean age at the time of amputation was 64 years (standard deviation±13.0) for women and 60 years (standard deviation±11.7) for men. Amputations were most frequent among individuals aged ≥65 years, with 1,473 procedures (40.7%). For both sexes, 91.0% of amputations occurred in residents of the Manaus intermediate geographic region, with 50.4% of amputations at the toe level (Table 1).

Age-adjusted amputation procedure rates increased among males, showing a single statistically significant upward trend, rising from 8.33 amputations per 100 thousand men in 2012 to 15.55 per 100 thousand men in 2021 (APC 10.5%; 95%CI 3.8; 17.6). Among women, age-adjusted amputation procedure rates rose from 4.80 per 100 thousand women in 2012 to 6.50 per 100 thousand women in 2021, without a statistically significant increase (APC 4.3%; 95%CI -5.0; 14.5) (Tables 2 and 3).

During the period 2017–2021, 2,549 diabetes-related amputations occurred, of which 1,749 were incident cases, with a mean reamputation rate of 45.7%. A total of 21,845.11 YLD were calculated for Amazonas (79.23 YLD per 100 thousand inhabitants/year). Men had a higher YLD (112.09 YLD per 100 thousand inhabitants/year) than women (47.44 YLD per 100 thousand inhabitants/year) for amputation-related disease burden. Most YLD were concentrated in the 45–64 years age group (58.2%). The burden of amputations was higher for men in all age groups compared to women (Table 4).

**Table 1:** Distribution of lower-limb amputations due to diabetes, according to sex, age group, intermediate geographic regions, and level of amputation. Amazonas, 2012–2021 (n=3,621)

Variable	Male	Female	Total
	n (%)	n (%)	n (%)
Amputations	2,465 (68.1)	1,156 (31.9)	3,621 (100.0)
**Age group** (years)			
<45	183 (7.4)	76 (6.6)	259 (7.2)
45–54	527 (21.4)	169 (14.6)	696 (19.2)
55–64	856 (34.7)	337 (29.2)	1,193 (32.9)
≥65 years	899 (36.5)	574 (49.6)	1,473 (40.7)
**Intermediate geographic regions** (n=3,608)^a^			
Lábrea	30 (1.2)	13 (1.1)	43 (1.2)
Manaus	2,242 (91.2)	1,040 (90.5)	3,282 (91.0)
Parintins	134 (5.4)	61 (5.3)	195 (5.4)
Tefé	53 (2.2)	35 (3.1)	88 (2.4)
**Type of amputation**			
Major lower-limb segments	896 (36.4)	487 (42.1)	1,383 (38.2)
Foot/tarsus	276 (11.2)	136 (11.8)	412 (11.4)
Toe	1,293 (52.4)	533 (46.1)	1,826 (50.4)

^a^Totals differ due to missing data on municipality of residence.

**Table 2:** Rates of lower-limb amputation procedures due to diabetes, age-specific and age-adjusted, per 100 thousand inhabitants, by year and sex. Amazonas, 2012–2021 (n=3,621)

Year	Age group (years)				Adjusted rate per 100 thousand inhabitants
	<45	45–54	55–64	≥65 years	
	Specific rate per 100 thousand inhabitants				
Male					
2012	0.59	17.78	46.65	76.14	8.33
2013	0.64	12.84	34.14	54.97	6.23
2014	0.51	16.50	33.57	46.64	6.02
2015	0.82	17.63	52.85	58.69	8.03
2016	1.06	27.47	62.32	92.46	11.22
2017	1.60	40.39	92.61	116.39	15.67
2018	1.16	31.89	103.78	155.70	16.97
2019	1.57	35.84	113.14	137.43	17.34
2020	1.37	41.38	91.78	132.53	16.24
2021	1.90	32.54	89.63	129.63	15.55
Female					
2012	0.53	4.60	18.37	62.60	4.80
2013	0.26	11.41	16.48	32.56	3.71
2014	0.06	4.28	21.62	26.66	2.91
2015	0.32	10.00	11.26	28.90	3.16
2016	0.25	8.50	32.33	35.13	4.45
2017	0.50	12.01	34.40	63.09	6.38
2018	0.56	12.08	42.06	93.28	8.25
2019	1.04	9.60	32.46	88.07	7.58
2020	0.67	7.28	47.94	63.65	6.84
2021	0.54	12.10	27.82	73.31	6.50

**Table 3:** Annual percent change (APC) and 95% confidence interval (95%CI) of age-adjusted rates of lower-limb amputation procedures due to diabetes. Amazonas, 2012–2021 (n=3,621)

Sex	APC (95%CI)
Male	10.5% (3.8; 17.6)
Female	4.3% (-5.0; 14.5)

**Table 4:** Years lived with disability (YLD) due to lower-limb amputations from diabetes, by age group and sex. Amazonas, 2017–2021 (n=1,749 incident cases, estimated based on the mean reamputation rate)

Age group (years)	Absolute number of YLD (%)	YLD rate per 100 thousand inhabitants
<45		
Men	1,697.23 (7.8)	10.50
Women	729.39 (3.3)	4.59
Both	2,426.62 (11.1)	7.57
45–54		
Men	4,036.17 (18.5)	217.58
Women	1,166.48 (5.3)	64.36
Both	5,202.64 (23.8)	141.86
55–64		
Men	5,449.80 (25.0)	480.10
Women	2,045.25 (9.4)	179.23
Both	7,495.05 (34.4)	329.27
≥**65 years**		
Men	4,196.75 (19.2)	487.13
Women	2,524.05 (11.5)	259.19
Both	6,720.80 (30.7)	366.19
**All ages**		
Men	15,379.95 (70.4)	76.86^a^
Women	6,465.16 (29.6)	32.65^a^
Both	21,845.11 (100.0)	54.87^a^
**All ages** (**adjusted YLD rate**)		
Men	15,379.95 (70.4)	112.09^b^
Women	6,465.16 (29.6)	47.44^b^
Both	21,845.11 (100.0)	79.23^b^

^a^Unadjusted YLD rate; ^b^Age-adjusted YLD rate.

## Discussion 

Between 2012 and 2021, an increasing trend in lower-limb amputation procedure rates was observed, with a high burden of diabetes-related lower-limb amputations, particularly among men and individuals aged 45–64 years during the 2017–2021 period. This finding suggests that diabetes was a determining factor in amputations in Amazonas, consistent with previous reports from Brazil (2,7,12).

A similar pattern has been described in other Brazilian states. In the state of São Paulo, an increase in diabetes-related lower-limb amputations occurred mainly in the male population, rising from 12% in 2009 to 18% in 2020 (p-value<0.001) (19). In Brazil, diabetes showed an increasing trend from 7% in 2008 to 27% in 2020 (p-value<0.001), predominantly among men, and is becoming the primary diagnosis in the Brazilian Unified Health System (*Sistema Único de Saúde*, SUS) for lower-limb amputations (20).

Although state and national trends indicate an increase in diabetes-related amputations, the global literature presents an opposite scenario. International studies indicate a decline in lower-limb amputation rates between 1982 and 2011 across several populations, including Israel, the United Kingdom, and Australia (21).

Evidence indicates that the weighted-average reamputation rate among individuals with diabetes-related lower-limb amputations is 20% within one year, 30% within three years, and 46% after five years. Previous amputation, level of amputation, and presence of comorbidities were risk factors associated with reulceration, reamputation, mortality, and impaired functional status (17).

In this study, the five-year reamputation average estimated by that review was used to estimate the incidence of amputations based on the number of procedures performed. More healthy life years lost due to diabetes-related amputation were observed among men. This result may be explained by the fact that men with diabetes undergoing amputation in Amazonas were younger than women, indicating that, upon amputation, they were further from reaching life expectancy, which contributed to more healthy life years lost due to amputation. Men accounted for the absolute majority of amputations, carrying the greatest disability burden. The protective hormonal role of estrogen in women was also highlighted, which may lead to differences in immune system function between sexes (22).

Men seek health services less frequently and, in general, have poorer perception of disease signs and symptoms (23). This may result in fewer opportunities for treatment and preventable hospitalizations by primary care (24), as well as poorer care indicators for men with diabetes (25). The results of this study corroborate previous studies on the burden of diabetes. In Santa Catarina, the burden of disease from diabetes-related lower-limb amputations between 2008 and 2013 was analyzed, and men also showed higher YLD (5,344.00 per 100 thousand) than women (2,566.40 per 100 thousand) (26).

The intermediate geographic region of Manaus, composed of 21 municipalities, accounted for 73% of Amazonas’ population in 2022 (13). It was the most urbanized and developed region of the state, with most medium- and high-complexity health services concentrated there (27). These characteristics directly influenced the spatial distribution of procedures and the results presented in this study, as 91% of diabetes-related lower-limb amputations occurred among residents of this region. 

Compared with other regions of Brazil, the North had the lowest rates of health service use in 2013 (28). The limitation in the organization and provision of services for effective health regions in Amazonas was associated with insufficient specialized services, scarce human resources, inadequate physical infrastructure, low income, and population dispersion, insufficient transportation availability, and the large geographical distances typical of the Amazon, which is difficult to access (27).

Brazil has undergone an epidemiological transition, largely driven by the decline in communicable diseases alongside the growing burden of noncommunicable diseases. The southern and southeastern states appear to be at more advanced stages of the epidemiological transition compared with the northern and northeastern states. Between 1990 and 2006, YLD due to diabetes in the country increased by 118%, rising from the 15th to the 11th leading cause of disability. Although health outcomes have improved in the North and Northeast, higher YLD rates persist in many of these states compared with those in the South and Southeast. The challenge lies in controlling the growing burden of chronic noncommunicable diseases and reducing regional health inequalities across the country (29).

It is essential to consider the differences in the distribution of the diabetes disease burden in Amazonas, given that it represents a growing morbidity burden and is in epidemiological transition, posing a challenge to the health of the Amazonas population. Studies on the burden of lower-limb amputations in other Brazilian states are imperative, considering that the healthy life years lost due to diabetes complications represent a concern for Brazilian public health.

The results of this study highlighted the growing need to improve strategies for preventing foot ulcers in individuals with diabetes, given the high and early mortality among those who underwent amputations, especially among people with diabetes (6,4). Assessing foot sensory function in individuals with diabetes is essential not only to determine the degree of functional impairment but also to implement a comprehensive approach to diabetes management and prevent complications (5).

Guidance by a multidisciplinary health team should take into account the social, economic, cultural, and environmental factors that affect the health–disease process of individuals and their families (26). The importance of health promotion programs is also emphasized, as well as interventions for healthy eating, regular physical activity, restriction of tobacco and alcohol consumption, obesity control, and longitudinal care in primary health care (30).

The exclusive use of SUS data limited the conclusions of this study, as only notifications from SIH/SUS were evaluated. However, the representativeness of the sample relative to the population of Amazonas was emphasized, as 87% of individuals in this state relied on public health services in 2019 (7).

The main limitation of this study was the potential for readmission for additional amputations, which necessitated an indirect estimate of amputation incidence for the YLD calculation, using the average reamputation rate reported in the literature. Thus, there is a possibility of selection bias if the reamputation rate differs significantly between Amazonas. 

Other limitations included: the absence of social and economic data on individuals, which are important in determining health status and use of health services; and the use of a single disability weight for all amputation levels in the YLD calculation. Although classifications exist that assign distinct weights to different levels of amputation, such categorizations consider factors such as the presence or absence and duration of postoperative treatment, variables that are not available in hospital admission authorizations (11,12).

The internal validity of the study is noteworthy, since the proper completion of the hospital admission authorization is a mandatory requirement for reimbursement of hospital care services, and this administrative–financial characteristic encourages detailed and standardized data recording.

Strengthening primary health care within the context of SUS for reducing diabetes-related lower-limb amputations should be considered based on integrated actions that include extended service hours, expansion of the Family Health Strategy (*Estratégia de Saúde da Família*, ESF) to uncovered areas, active search for individuals at risk for or diagnosed with diabetes in the territory, guaranteed availability of hypoglycemic medications in the public network, and health education, promoting access, early diagnosis, and prevention of complications.

In conclusion, a higher burden and an increasing trend of diabetes-related lower-limb amputations among men in Amazonas were observed.

## Data Availability

The database is fully available at: https://data.mendeley.com/datasets/gvr4gdbjr3/1 (15).
