# Supplementary material for: Trend and burden of lower-limb amputations due to diabetes: time series analysis, Amazonas, 2012–2021
Source: Epidemiol Serv Saude. 2026 Mar 16;35:e20250534. doi: 10.1590/S2237-96222026v35e20250534.en (PMC12995277; doi:10.1590/S2237-96222026v35e20250534.en)
Supplement: Translation

# Translation: Tendência e carga de amputações de membros inferiores por diabetes: análise de série temporal, Amazonas, 2012-2021

**DOI:** 10.1590/S2237-96222026v35e20250534.pt

**Authors:** Cilio Antonio Ribeiro, Sabrina da Silva Santos, Thatiana Lameira Maciel Amaral

**Affiliations:** 1 Fundação Oswaldo Cruz, Escola Nacional de Saúde Pública Sergio Arouca, Rio de Janeiro, RJ, Brasil; 2 Universidade Federal do Acre, Rio Branco, AC, Brasil

**Keywords:** Diabetes Mellitus, Amputação Cirúrgica, Estudos de Séries Temporais, Carga Global da Doença, Anos de Vida Ajustados por Deficiência

## Abstract

**Objetivo:**

Calcular a tendência temporal e a carga da doença para as amputações de membros inferiores por diabetes no Amazonas, Brasil, no período 2012-2021.

**Métodos:**

Trata-se de estudo de análise de série temporal com dados do Sistema de Informações Hospitalares do Sistema Único de Saúde. Calcularam-se as taxas específicas e ajustadas por idade, a variação percentual anual das amputações e o intervalo de confiança de 95% no período 2012-2021. Foram calculados os anos vividos com incapacidade estratificados por sexo e faixa etária entre 2017 e 2021.

**Resultados:**

Durante os 10 anos analisados, ocorreram 3.621 procedimentos de amputações de membros inferiores em pessoas com diabetes nos hospitais públicos do Amazonas. Verificou-se aumento das taxas de amputação ajustadas por idade em homens, passando de 8,33 em 2012 para 15,55 por 100 mil homens em 2021, com variação percentual anual de 10,5% e intervalo de confiança de 95% entre 3,8% e 17,6%. A carga da doença totalizou 21.845,11 anos vividos com incapacidade, dos quais 70,4% corresponderam aos homens. A maior parte dos anos vividos com incapacidade se concentrou entre 45-64 anos (58,2%).

**Conclusão:**

Houve predominância masculina na tendência crescente das amputações de membros inferiores e na alta carga da doença no Amazonas.

**Aspectos éticos**

Esta pesquisa utilizou bancos de dados de domínio público, anonimizados e com acesso irrestrito no Departamento de Informática do Sistema Único de Saúde.:

## Introdução 

As doenças crônicas não transmissíveis constituem a principal causa de morbidade e mortalidade no mundo, com maior efeito nas populações mais vulnerabilizadas que vivem em países de baixa e média renda (1). Entre essas doenças, o diabetes apresenta elevado custo econômico e social às pessoas e aos sistemas de saúde, uma vez que a doença está associada a complicações crônicas de diferentes gravidades, que comprometem a capacidade funcional, reduzem a autonomia e impactam a qualidade de vida (2,3).

As complicações dos membros inferiores relacionadas ao diabetes frequentemente iniciam com neuropatia, um fator de risco crítico para o desenvolvimento de ulceração, que, por sua vez, favorece infecções e amputações (4,5). Cerca de 90% das amputações não traumáticas de membros inferiores foram atribuídas ao diabetes entre 2003 e 2013. Globalmente, a cada 30 segundos, um membro inferior é amputado em decorrência de complicações associadas à doença; e mais de 50% das pessoas com diabetes morrem no período de cinco anos após a primeira amputação (4,6).

Em 2019, 606.785 adultos com diabetes (6%) relataram úlceras ou amputações no Brasil (7). No Amazonas, a incidência de diabetes foi quase duas vezes maior entre mulheres (165,23/100 mil habitantes) em comparação aos homens (93,72/100 mil habitantes) em 2013. No entanto, as complicações crônicas mais graves, como as amputações de membros inferiores, apresentaram incidências mais elevadas no sexo masculino (4,99/100 mil habitantes) em relação ao feminino (2,75/100 mil habitantes). 

No Brasil e no Amazonas, 55% das pessoas com diabetes nunca tiveram seus pés examinados por um profissional de saúde. A ausência de acompanhamento contínuo também se evidenciou no monitoramento do controle glicêmico, com 10% das pessoas com diabetes do país, em 2013, convivendo com a doença há mais de um ano sem medição regular da glicemia. No Amazonas, esse quantitativo subiu para 12% (8). Essas fragilidades no cuidado e a divergência no perfil epidemiológico entre homens e mulheres reforçam a importância de investigações específicas ao contexto local e de análises estratificadas por sexo.

A mensuração do estado de saúde das populações, diante do aumento da expectativa de vida e da elevada prevalência de doenças crônicas não transmissíveis, deve considerar a tendência temporal e o tempo vivido com alguma doença (1,9). A Organização Mundial da Saúde implementou o cálculo dos anos de vida vividos com incapacidade, como importante indicador da magnitude da carga global da doença, no intuito de mensurar a repercussão da morbidade na saúde das populações, sensível às disparidades em saúde e útil para identificar grupos vulnerabilizados (10). Em 2021, apontou-se que os anos de vida vividos com incapacidade devido ao diabetes foi crescente em todos os países, com aumento global de 26% entre 2010 e 2021 (11,12).

Este artigo teve como objetivo calcular a tendência temporal e a carga da doença para as amputações de membros inferiores por diabetes da população geral do Amazonas no período 2012-2021.

## Métodos 

### Delineamento do estudo

Trata-se de estudo de análise de série temporal e carga da doença para as amputações de membros inferiores por diabetes na população geral do Amazonas. Utilizou-se o indicador de anos vividos com incapacidade (*years lived with disability* – YLD), medida que visou apreender o efeito da morbidade no estado de saúde de populações.

### Contexto 

O Amazonas está localizado na região Norte e é o maior estado do país em extensão territorial, com área de 1.559.255,881 km^2^. Conforme o Censo Demográfico 2022 do Instituto Brasileiro de Geografia e Estatística (IBGE), a população do estado atingiu 3.941.613 habitantes em 2022. A densidade demográfica era de 2,53 habitantes/km^2^, colocando-o como o segundo estado mais populoso do Norte (13). 

O Amazonas é composto por 62 municípios. Para fins analíticos, este estudo adotou a estratificação geográfica com base nas quatro regiões geográficas intermediárias definidas pelo IBGE: Lábrea, Manaus, Parintins e Tefé.

### Participantes 

O estudo abrangeu a população geral do Amazonas, de ambos os sexos e de todas as idades, no período 2012-2021. Nessa população, foi identificado o número total de amputações de indivíduos com diabetes, com base no município de residência das pessoas amputadas, registrado no banco de dados do Sistema de Informações Hospitalares do Sistema Único de Saúde.

### Variáveis e fonte de dados

As informações sobre amputações foram obtidas do Sistema de Informações Hospitalares do Sistema Único de Saúde (14), a partir das autorizações de internações hospitalares de indivíduos submetidos ao procedimento de amputação devido ao diabetes, no Amazonas, no período 2012-2021, disponíveis no Departamento de Informática do Sistema Único de Saúde. 

Os dados necessários para identificar o número total de procedimentos de amputação foram: diagnóstico primário (Classificação Internacional de Doenças – versão 10: E10-E14), sexo, faixa etária na data da amputação, município de residência no Amazonas para determinação das regiões geográficas intermediárias e procedimentos realizados segundo nível de amputação (membros inferiores maiores, pé/tarso, dedo) (15).

As populações de homens e mulheres residentes no Amazonas, por faixa-etária, foram obtidas da base de dados do Departamento de Informática do Sistema Único de Saúde (https://datasus.saude.gov.br/), utilizando o estudo de estimativas populacionais por município, idade e sexo (16) para o período 2012-2021. Essas populações foram utilizadas no cálculo das taxas de procedimentos de amputações.

### Viés 

Os dados coletados referiram-se às autorizações de internações hospitalares para cirurgias de amputação devido ao diabetes. As distribuições e as taxas de amputações apresentadas devem ser interpretadas como número e magnitude dos procedimentos realizados, e não como número de indivíduos submetidos às amputações e taxas de incidência.

Para o cálculo do YLD, utilizaram-se estimativas de casos incidentes. Diante da ausência desse parâmetro para o Amazonas, estimou-se a incidência de amputações considerando a média de reamputações de 45,7% (17). O número de autorizações de internações hospitalares foi dividido por 1,457.

### Métodos estatísticos

Os dados foram organizados em planilhas eletrônicas no Microsoft Excel e foram apresentados como valores absolutos e proporcionais segundo sexo, faixa etária e regiões geográficas intermediárias do Amazonas.

Para a tendência temporal, foram calculadas taxas, específicas e padronizadas por idade, das amputações de membros inferiores por diabetes. Para essa padronização por idade, utilizou-se o método direto, tendo como população padrão a média da população de homens e mulheres do Amazonas entre 2012 e 2021 (16). Esse período foi selecionado por oferecer uma série histórica consistente, com dados mais padronizados no Sistema de Informações Hospitalares a partir de 2012, o que aumentou a comparabilidade e a confiabilidade dos registros.

O Joinpoint Trend Analysis Software, versão 4.9.1.0 (disponível em https://surveillance.cancer.gov/joinpoint/), foi empregado para determinar a variação percentual anual (*annual percent change* – APC) e o intervalo de confiança de 95% (IC95%) das taxas de amputações padronizadas por idade, que foram calculadas pelo programa. Os parâmetros considerados na análise de tendência incluíram: taxas como variável dependente, ano como variável independente, mínimo 0 e máximo 5 pontos de junção, ajuste dos erros por autocorrelação de primeira ordem calculada a partir dos dados, critério de informação bayesiana ponderada para seleção do modelo final e nível de significância de 5%.

Para a análise de carga da doença associada à amputação por diabetes, calculou-se o YLD, para o período 2017-2021, da seguinte forma: YLD=IxPxD, em que I correspondeu ao total de casos incidentes de amputações; P referiu-se ao peso da incapacidade; e D compreendeu a duração média da incapacidade permanente resultante da amputação (considerada até o final da expectativa de vida do indivíduo) (10). 

Considerou-se a expectativa de vida ao nascer de 89,95 anos (idade esperada para o óbito), conforme a tabela de referência de risco mínimo teórico de vida do estudo sobre Carga Global de Doença de 2021 (12). O peso da incapacidade utilizado neste estudo foi 0,36, que correspondeu ao peso considerado para amputações, segundo Murray e Lopez (18). Estes quantificaram a perda de saúde ocorrida durante o tempo vivido com a doença, variando de 0 (estado de saúde plena) a 1 (pior grau, equivalente à morte).

Foram apresentados os valores absolutos e proporcionais do YLD. Um YLD representou um ano de vida saudável perdido devido à incapacidade (10). Apresentaram-se as taxas brutas de YLD por 100 mil homens ou mulheres da população do Amazonas, para cada faixa etária, e as taxas de YLD ajustadas por idade, tendo como padrão a população mundial do estudo sobre Carga Global de Doença de 2021 (11).

## Resultados 

Durante os 10 anos analisados (2012-2021), ocorreram 3.621 amputações de membros inferiores nos hospitais públicos do Amazonas, distribuídas em 4 regiões geográficas intermediárias. A maior parte das amputações ocorreu em homens (68,1%) (Tabela 1).

A média de idade registrada nas amputações de mulheres foi 64 anos (desvio-padrão±13,0) e de homens, 60 anos (desvio-padrão±11,7). As amputações foram mais frequentes entre indivíduos ≥65 anos, com 1.473 (40,7%) procedimentos. Para ambos os sexos, 91,0% de amputações ocorreram em residentes da região geográfica intermediária de Manaus, com 50,4% de amputações ao nível dos dedos dos pés (Tabela 1).

Houve aumento das taxas de procedimentos de amputação ajustadas por idade no sexo masculino, que manteve uma única tendência de crescimento estatisticamente significativo, passando de 8,33 amputações/100 mil homens em 2012 para 15,55/100 mil homens em 2021 (APC 10,5%; IC95% 3,8; 17,6). Entre as mulheres, as taxas de procedimentos de amputação ajustadas por idade passaram de 4,80/100 mil mulheres em 2012 para 6,50/100 mil mulheres em 2021, não sendo observado aumento estatisticamente significativo (APC 4,3%; IC95% -5,0; 14,5) (Tabelas 2 e 3).

No período 2017-2021, ocorreram 2.549 amputações por diabetes, a partir das quais estimaram-se 1.749 casos incidentes, considerando a média de reamputações de 45,7%. Foram calculados para o Amazonas 21.845,11 YLD (79,23 YLD por 100 mil habitantes/ano). Os homens manifestaram maior YLD (112,09 YLD por 100 mil habitantes/ano) do que as mulheres (47,44 YLD por 100 mil habitantes/ano) na comparação da carga da doença por amputações entre os sexos. A maior parte do YLD concentrou-se na faixa etária 45-64 anos (58,2%). A carga de amputações foi maior para os homens em todas as faixas etárias em comparação com as mulheres (Tabela 4).

**Tabela 1:** Distribuição das amputações de membros inferiores por diabetes, segundo sexo, faixa etária, regiões geográficas intermediárias e nível da amputação. Amazonas, 2012-2021 (n=3.621)

Variável	Masculino	Feminino	Total
	n (%)	n (%)	n (%)
Amputações	2.465 (68,1)	1.156 (31,9)	3.621 (100,0)
**Faixa etária** (anos)			
<45	183 (7,4)	76 (6,6)	259 (7,2)
45-54	527 (21,4)	169 (14,6)	696 (19,2)
55-64	856 (34,7)	337 (29,2)	1.193 (32,9)
≥65 anos	899 (36,5)	574 (49,6)	1.473 (40,7)
**Regiões geográficas intermediárias** (n=3.608)^a^			
Lábrea	30 (1,2)	13 (1,1)	43 (1,2)
Manaus	2.242 (91,2)	1.040 (90,5)	3.282 (91,0)
Parintins	134 (5,4)	61 (5,3)	195 (5,4)
Tefé	53 (2,2)	35 (3,1)	88 (2,4)
**Nível da amputação**			
Membros inferiores maiores	896 (36,4)	487 (42,1)	1.383 (38,2)
Pé/tarso	276 (11,2)	136 (11,8)	412 (11,4)
Dedo	1.293 (52,4)	533 (46,1)	1.826 (50,4)

^a^Os totais divergem devido a dados faltantes de municípios de residência.

**Tabela 2:** Taxas de procedimentos de amputações de membros inferiores por diabetes, específicas por faixa etária e ajustadas por idade, por 100 mil habitantes, segundo ano e sexo. Amazonas, 2012-2021 (n=3.621)

Ano	Faixa etária (anos)				Taxa ajustada por 100 mil habitantes
	<45	45-54	55-64	≥65 anos	
	Taxa específica por 100 mil habitantes				
Masculino					
2012	0,59	17,78	46,65	76,14	8,33
2013	0,64	12,84	34,14	54,97	6,23
2014	0,51	16,50	33,57	46,64	6,02
2015	0,82	17,63	52,85	58,69	8,03
2016	1,06	27,47	62,32	92,46	11,22
2017	1,60	40,39	92,61	116,39	15,67
2018	1,16	31,89	103,78	155,70	16,97
2019	1,57	35,84	113,14	137,43	17,34
2020	1,37	41,38	91,78	132,53	16,24
2021	1,90	32,54	89,63	129,63	15,55
Feminino					
2012	0,53	4,60	18,37	62,60	4,80
2013	0,26	11,41	16,48	32,56	3,71
2014	0,06	4,28	21,62	26,66	2,91
2015	0,32	10,00	11,26	28,90	3,16
2016	0,25	8,50	32,33	35,13	4,45
2017	0,50	12,01	34,40	63,09	6,38
2018	0,56	12,08	42,06	93,28	8,25
2019	1,04	9,60	32,46	88,07	7,58
2020	0,67	7,28	47,94	63,65	6,84
2021	0,54	12,10	27,82	73,31	6,50

**Tabela 3:** Variação percentual anual (*annual percent change* – APC) e intervalo de confiança de 95% (IC95%) das taxas de procedimentos de amputações de membros inferiores por diabetes ajustadas por idade. Amazonas, 2012-2021 (n=3.621)

Sexo	APC (IC95%)
Masculino	10,5% (3,8; 17,6)
Feminino	4,3% (-5,0; 14,5)

**Tabela 4:** Anos vividos com incapacidade (*years lived with disability* – YLD) para as amputações de membros inferiores por diabetes, segundo faixa etária e sexo. Amazonas, 2017-2021 (n=1.749 casos incidentes, estimados pela média de reamputações)

Faixa etária (anos)	Número absoluto de YLD (%)	Taxa de YLD por 100 mil habitantes
<45		
Homens	1.697,23 (7,8)	10,50
Mulheres	729,39 (3,3)	4,59
Ambos	2.426,62 (11,1)	7,57
45-54		
Homens	4.036,17 (18,5)	217,58
Mulheres	1.166,48 (5,3)	64,36
Ambos	5.202,64 (23,8)	141,86
55-64		
Homens	5.449,80 (25,0)	480,10
Mulheres	2.045,25 (9,4)	179,23
Ambos	7.495,05 (34,4)	329,27
≥**65 anos**		
Homens	4.196,75 (19,2)	487,13
Mulheres	2.524,05 (11,5)	259,19
Ambos	6.720,80 (30,7)	366,19
**Todas as idades**		
Homens	15.379,95 (70,4)	76,86^a^
Mulheres	6.465,16 (29,6)	32,65^a^
Ambos	21.845,11 (100,0)	54,87^a^
**Todas as idades** (**taxa de YLD ajustada**)		
Homens	15.379,95 (70,4)	112,09^b^
Mulheres	6.465,16 (29,6)	47,44^b^
Ambos	21.845,11 (100,0)	79,23^b^

^a^Taxa bruta de YLD; ^b^Taxa de YLD ajustada por idade.

## Discussão 

Entre 2012 e 2021, verificou-se tendência crescente da taxa de procedimentos de amputação, com alta carga de amputações dos membros inferiores por diabetes, sobretudo em homens e na faixa etária 45-64 anos no período 2017-2021. Isso sugeriu que o diabetes foi fator determinante para amputações no Amazonas, assim como previamente reportado para o Brasil (2,7,12).

Padrão semelhante foi descrito em outros estados brasileiros. No estado de São Paulo, ocorreu aumento de amputações de membros inferiores relacionadas ao diabetes, principalmente na população masculina, de 12% em 2009 para 18% em 2020 (p-valor<0,001) (19). No Brasil, o diabetes apresentou tendência crescente de 7% em 2008 para 27% em 2020 (p-valor<0,001), predominantemente em homens, e está se tornando o diagnóstico primário do Sistema Único de Saúde para amputações de membros inferiores (20).

Embora as tendências estaduais e nacional indiquem aumento nas amputações relacionadas ao diabetes, a literatura global apresenta um quadro contrário. Estudos internacionais indicam tendência de redução nas taxas de amputação de membros inferiores, entre 1982 e 2011, em diversas populações, como em Israel, no Reino Unido e na Austrália (21).

Evidências indicaram que a média ponderada de reamputação em pessoas com amputações de membros inferiores por diabetes é de 20% em um ano, 30% em três anos e 46% após cinco anos. A ocorrência de amputações anteriores, o nível da amputação e a presença de comorbidades constituíram fatores de risco associados à reulceração, à reamputação, à mortalidade e ao comprometimento do estado funcional (17).

Neste estudo, considerou-se a média de reamputações em cinco anos estimada por essa revisão para estimar a incidência de amputações a partir do número de procedimentos realizados. Verificaram-se mais anos de vida saudável perdidos pela amputação por diabetes em homens. Esse resultado pode ser justificado devido aos homens com diabetes amputados do Amazonas serem mais jovens que as mulheres, indicando que, ao sofrerem a amputação, estavam mais distantes de alcançar a expectativa de vida, o que contribuiu para mais anos de vida saudável perdidos pela amputação. Os homens representaram a maioria absoluta das amputações, carregando o fardo de maior carga de incapacidade. Enfatizou-se ainda o papel protetor hormonal do estrogênio nas mulheres, que pode levar a diferenças na função do sistema imunológico entre os sexos (22).

Os homens procuram com menor frequência os serviços de saúde e, em geral, apresentam pior percepção de sinais e sintomas de doenças (23). Isso pode resultar em menor oportunidade de tratamentos e de internações preveníveis pela atenção primária (24), bem como verificar piores indicadores de cuidado assistencial para homens com diabetes (25). Os resultados deste estudo corroboram estudos anteriores sobre carga do diabetes. Em Santa Catarina, foi analisada a carga da doença para as amputações de membros inferiores por diabetes entre 2008 e 2013: os homens também apresentaram maior YLD (5.344,00/100 mil) em comparação com as mulheres (2.566,40/100 mil) (26).

A região geográfica intermediária de Manaus, composta por 21 municípios, concentrava 73% da população do Amazonas em 2022 (13). Tratava-se da região mais urbanizada e desenvolvida do estado, centralizando a maior parte dos serviços de saúde de média e alta complexidade (27). Essas características influenciaram diretamente a distribuição espacial dos procedimentos e resultados apresentados neste estudo, uma vez que 91% dos procedimentos de amputações de membros inferiores por diabetes ocorreram em residentes dessa região. 

Em comparação às demais regiões do Brasil, o Norte abrangeu os menores índices de utilização dos serviços de saúde em 2013 (28). A limitação na organização e prestação de serviços para efetivas regiões de saúde no Amazonas estava associada a serviços especializados insuficientes, recursos humanos escassos, infraestruturas físicas inadequadas, baixa renda e dispersão populacional, disponibilidade de transporte insuficiente e grandes distâncias geográficas típicas da Amazônia de difícil acesso (27).

O Brasil sofreu uma transição epidemiológica, em grande parte atribuível ao declínio das doenças transmissíveis que ocorreu paralelamente à crescente carga de doenças não transmissíveis. Os estados do Sul e do Sudeste parecem estar em estágios avançados da transição epidemiológica em comparação com os estados do Norte e Nordeste. Entre 1990 e 2006, os YLD devido ao diabetes no país cresceram 118%, passando da 15ª para a 11ª principal causa de incapacidade. Embora os resultados de saúde tenham melhorado nos estados do Norte e Nordeste, as taxas mais altas de YLD persistem em muitos estados dessas regiões em comparação com os estados do Sul e Sudeste. O desafio está em controlar a crescente carga de doenças crônicas não transmissíveis e diminuir as desigualdades regionais de saúde existente no país (29).

É essencial considerar as diferenças no perfil da distribuição da carga da doença do diabetes para o Amazonas, visto que se trata de carga de morbidade crescente e em transição epidemiológica, representando um desafio para a saúde da população amazonense. São imprescindíveis estudos de carga de amputações de membros inferiores em outros estados brasileiros, tendo em vista que os anos de vida saudável perdidos devido às complicações do diabetes representam uma preocupação para a saúde pública brasileira.

Os resultados deste estudo destacaram a crescente necessidade de aprimorar estratégias de prevenção de úlceras no pé de uma pessoa com diabetes, dada a elevada e precoce mortalidade entre os indivíduos que sofreram amputações, especialmente entre as pessoas com diabetes (6,4). A análise da função sensorial dos pés em indivíduos com diabetes é essencial não apenas para determinar o grau de comprometimento funcional, mas também para implementar uma abordagem integral ao manejo do diabetes e prevenir complicações (5).

A orientação por parte de uma equipe de saúde multidisciplinar deve levar em consideração os fatores sociais, econômicos, culturais e ambientais que afetam o processo saúde-doença das pessoas e suas famílias (26). Destaca-se, ainda, a importância de programas de promoção à saúde, intervenções para alimentação saudável, prática regular de atividade física, restrição de tabaco e consumo de álcool, controle da obesidade e cuidados longitudinais na atenção primária em saúde (30).

O uso exclusivo de dados do Sistema Único de Saúde restringiu as conclusões deste estudo, tendo em vista que foram avaliadas somente notificações do Sistema de Informações Hospitalares. Todavia, enfatizou-se a representatividade da amostra em relação à população do Amazonas, uma vez que 87% dos indivíduos desse estado dependiam dos serviços de saúde pública em 2019 (7).

A principal limitação deste estudo foi a possibilidade de reinternação para amputações adicionais, o que gerou a necessidade de estimativa indireta da incidência de amputações para o cálculo do YLD, por meio da média de reamputações descrita na literatura. Assim, existe a possibilidade de viés de seleção, caso a ocorrência de reamputações seja significativamente diferente no Amazonas. 

Outras limitações incluíram: ausência de dados sociais e econômicos dos indivíduos, importantes na determinação do estado de saúde e do uso dos serviços de saúde; e utilização de um peso único para todos os níveis de amputação no cálculo do YLD. Embora existam classificações que atribuem pesos distintos aos diferentes níveis de amputação, essa categorização considerou aspectos como a presença ou ausência e o tempo de tratamento do indivíduo amputado, variáveis que não estão disponíveis nas autorizações de internações hospitalares (11,12).

Ressalta-se a validade interna do estudo, uma vez que o preenchimento adequado da autorização de internação hospitalar é requisito obrigatório para o reembolso dos serviços de assistência hospitalar e este caráter administrativo-financeiro incentiva o registro detalhado e padronizado dos dados.

O fortalecimento da atenção primária em saúde no contexto do Sistema Único de Saúde para a redução de amputações de membros inferiores por diabetes deve ser considerado a partir de ações integradas que incluam ampliação de horários de atendimento, expansão da Estratégia de Saúde da Família para áreas descobertas, busca ativa de indivíduos com risco ou diagnóstico de diabetes no território, garantia de hipoglicemiantes na rede pública e educação em saúde, favorecendo o acesso, o diagnóstico precoce, a prevenção de complicações.

Em conclusão, observou-se maior carga e tendência crescente de amputações de membros inferiores por diabetes entre homens no Amazonas.

## Data Availability

O banco de dados está disponível integralmente em: https://data.mendeley.com/datasets/gvr4gdbjr3/1 (15).
